# Preoperative systemic inflammation response index is an independent prognostic marker for BCG immunotherapy in patients with non‐muscle‐invasive bladder cancer

**DOI:** 10.1002/cam4.5284

**Published:** 2022-10-10

**Authors:** Kun Ye, Ming Xiao, Zitaiyu Li, Kancheng He, Jinhua Wang, Liang Zhu, Wei Xiong, Zhaohui Zhong, Yuxin Tang

**Affiliations:** ^1^ Department of Urology The Fifth Affiliated Hospital of Sun Yat‐Sen University Zhuhai Guangdong China; ^2^ Guangdong Provincial Key Laboratory of Biomedical Imaging The Fifth Affiliated Hospital, Sun Yat‐Sen University Zhuhai Guangdong China; ^3^ Department of Urology The Second Xiangya Hospital, Central South University Changsha Hunan China

**Keywords:** BCG response, biomarkers, inflammation, NMIBC, systemic inflammatory response index

## Abstract

**Background:**

The Systemic Inflammatory Response Index (SIRI) is a novel prognostic biomarker based on peripheral blood counts of neutrophils, monocytes, and lymphocytes. Recent evidence suggests that it is associated with poor prognosis in various cancers. However, the predictive value of the SIRI in non‐muscle‐invasive bladder cancer (NMIBC) patients treated with intravesical Bacillus Calmette‐Guerin (BCG) immunotherapy remains elusive. Therefore, this study aimed to evaluate the potential of SIRI as a prognostic factor in these patients.

**Methods:**

A total of 540 patients with NMIBC who underwent BCG immunotherapy following transurethral resection of bladder tumor (TURBT) were enrolled in this study. Using receiver operating characteristic (ROC) curves and the Youden index, patients were divided into high and low SIRI groups based on the cutoff values. Univariable and multivariable logistic regression analyses were performed to identify independent predictors of BCG non‐response. Thereafter, propensity score matching (PSM) was used to eliminate bias due to confounding factors between the low and high SIRI groups. Finally, the Kaplan–Meier method was used to compare recurrence‐free survival (RFS) and progression‐free survival (PFS) between the two groups.

**Results:**

Multivariable logistic regression analysis revealed that high SIRI (*p* = 0.001), high MLR (*p* = 0.015), and high tumor pathological T stage (*p* = 0.015) were significantly correlated with non‐response to BCG therapy. In addition, both RFS and PFS were shorter in the high SIRI group than in the other group before and after PSM (both *p* < 0.05). Collectively, our results indicate that the combination of tumor pathological T staging and the SIRI can enhance the predictive power of BCG response.

**Conclusion:**

Pretreatment peripheral blood SIRI can be employed to predict the response to BCG immunotherapy and the prognosis of NMIBC patients. Taken together, the combination of T stage and SIRI demonstrated robust performance in predicting the response to BCG immunotherapy in NMIBC patients.

## Graphical abstract

The Systemic Inflammatory Response Index (SIRI) is a novel prognostic biomarker based on peripheral blood counts of neutrophils, monocytes, and lymphocytes. However, the predictive value of the SIRI in non‐muscle‐invasive bladder cancer (NMIBC) patients treated with intravesical Bacillus Calmette‐Guerin (BCG) immunotherapy remains elusive. Therefore, this study aimed to evaluate the potential of SIRI as a prognostic factor in these patients. A total of 540 patients with NMIBC who underwent BCG immunotherapy following transurethral resection of bladder tumor (TURBT) were enrolled in this study. Using receiver operating characteristic (ROC) curves and the Youden index, patients were divided into high and low SIRI groups based on the cutoff values. Univariable and multivariable logistic regression analyses were performed to identify independent predictors of BCG non‐response. Thereafter, propensity score matching (PSM) was used to eliminate bias due to confounding factors between the low and high SIRI groups. Finally, the Kaplan‐Meier method was used to compare recurrence‐free survival (RFS) and progression‐free survival (PFS) between the two groups. Multivariable logistic regression analysis revealed that high SIRI (*p* = 0.001), high MLR (*p* = 0.015), and high tumor pathological T stage (*p* = 0.015) were significantly correlated with non‐response to BCG therapy. In addition, both RFS and PFS were shorter in the high SIRI group than in the other group before and after PSM (both *p* < 0.05). Pretreatment peripheral blood SIRI can be employed to predict the response to BCG immunotherapy and the prognosis of NMIBC patients. The combination of T stage and SIRI demonstrated robust performance in predicting the response to BCG immunotherapy in NMIBC patients.

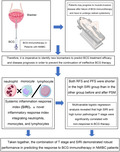

## 1 INTRODUCTION

Bladder cancer is one of the most common malignancies of the genitourinary system and is divided into the following two main types^1^: Non‐muscle‐invasive bladder cancer (NMIBC) and muscle‐invasive bladder cancer (MIBC). The former includes Ta and T1 stages, whereas the latter comprises T2, T3, and T4 stages.^2^ Pathologically, bladder cancer includes urothelial carcinoma and other histological types (squamous cell carcinoma, adenocarcinoma, small cell carcinoma, etc.), with urothelial carcinoma accounting for 75% of cases.^3^ At present, the treatment of NMIBC is predominantly based on transurethral resection of bladder tumor (TURBT), followed by intravesical instillation therapy (including chemotherapy and immunotherapy), with Bacille Calmette‐Guérin (BCG) being the most commonly used drug for immunotherapy.^4^ However, up to 80% of patients might suffer from disease recurrence after treatment, and approximately 40% of them might progress to muscle‐invasive disease. The latest EAU guidelines recommend intravesical BCG immunotherapy for high‐risk NMIBC patients.^5^ However, its efficacy therapy remains limited, and patients with disease recurrence and progression often have to undergo radical cystectomy (RC). Therefore, it is imperative to identify new biomarkers to predict BCG treatment efficacy and disease prognosis in order to prevent the continuation of ineffective BCG therapy.

A previous study indicated that the inflammatory reaction and immune surveillance play a critical role in tumor initiation, progression, and prognosis and that patients can be distinguished by alterations in the number of immune cells in the peripheral blood.^6^ Different clinical indicators in the peripheral blood, such as the absolute counts of monocytes, lymphocytes, and neutrophils, have been explored as promising prognostic indicators in different tumor models, while others have been converted into ratios, such as the platelet‐to‐lymphocyte ratio (PLR), neutrophil‐to‐lymphocyte ratio (NLR), and monocyte‐to‐lymphocyte ratio (MLR).^7^, ^8^, ^9^, ^10^ As is well‐documented, low lymphocyte counts and high neutrophil and/or monocyte counts may impact prognosis in various types of cancer.

To date, studies on the predictive value of inflammatory markers in NMIBC patients receiving intravesical BCG immunotherapy are scarce. In this study, we thus evaluated a novel inflammatory response index that integrated neutrophils, monocytes, and lymphocytes, termed the systemic inflammation response index (SIRI), which has been reported to have a potential predictive role in different cancers.^11^, ^12^ Considering that there was no report on the use of the SIRI in NMIBC patients undergoing intravesical BCG immunotherapy, this study aimed to assess its prognostic value in this patient population.

## 2 MATERIALS AND METHODS

### 2.1 Patient selection

**FIGURE 1:**
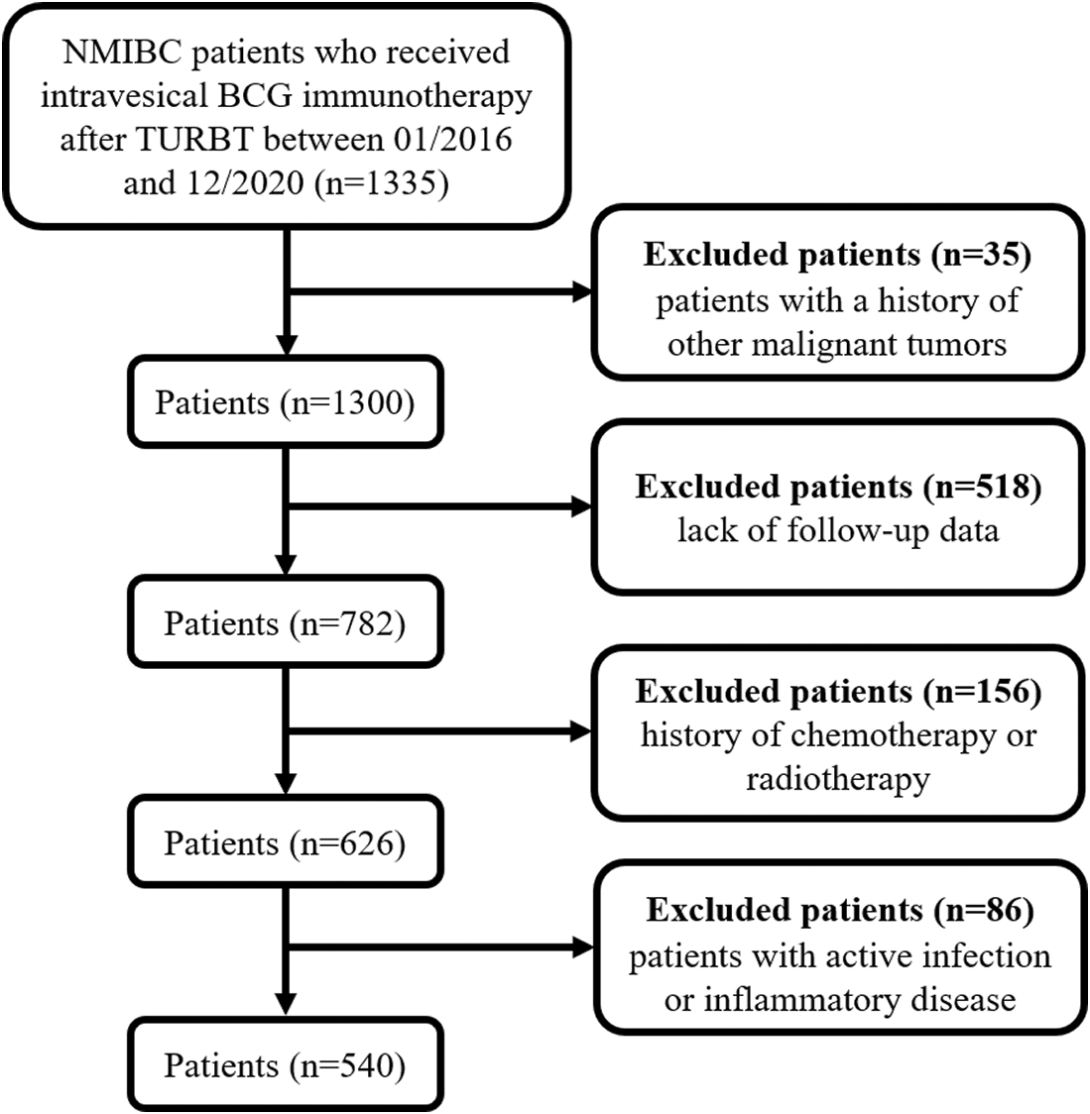
Diagram of the study population

This retrospective study included a total of 1335 NMIBC patients who underwent intravesical BCG immunotherapy following transurethral resection of bladder tumor (TURBT) between January 2016 and December 2020 at the Second Xiangya Hospital of Central South University. The inclusion criteria were as follows: (1) patients aged >18 years, (2) pathological examination confirmed the diagnosis of bladder urothelial carcinoma without muscle invasion, (3) patients that have undergone intravesical BCG instillation after TURBT, (4) BCG therapy was well‐tolerated without any treatment interruption, and (5) complete baseline characteristics were available. The exclusion criteria were as follows: (1) history of other malignant tumors (*n* = 35), (2) lack of follow‐up data (*n* = 518), (3) history of chemotherapy or radiotherapy (*n* = 156), and (4) active infection or inflammatory disease (*n* = 86). Ultimately, this study enrolled 540 patients. A flowchart of the population is illustrated in Figure 1. This retrospective study was approved by the Ethics Committee of the Second Xiangya Hospital of Central South University and was performed in accordance with the Declaration of Helsinki. Owing to the retrospective nature of this study and the patient data being anonymized and deidentified prior to analysis, written informed consent was not required.

### 2.2 Clinical variables

The variables collected in this study were as follows: age, sex, smoking status, diabetes status, hypertension status, tumor recurrence, tumor progression, BCG response, and primary tumor characteristics (pathological T stage, pathological grade, lymphovascular infiltration (LVI), carcinoma in situ (CIS), tumor number, and tumor size). Hematological data, such as neutrophil, monocyte, and lymphocyte counts, were acquired following blood collection within 3 days before surgery to calculate the PLR, NLR, MLR, and SIRI. PLR, NLR, MLR, and SIRI were calculated using the following formulas: PLR = platelet count/lymphocyte count, NLR = neutrophil count/lymphocyte count, MLR = monocyte count/lymphocyte count, SIRI = neutrophil count × monocyte count/lymphocyte count.

### 2.3 Patient follow‐up

Discharged patients were closely followed up to monitor disease recurrence, progression, and BCG response. Patient follow‐up included cystoscopy, blood and urine cytology, and abdominal computed tomography (CT). The BCG immunotherapy schedule was as follows: 120 mg weekly during the initial induction period for a total of 6 times, then 120 mg once every 2 weeks for a total of 3 intensive cycles, and finally, a monthly maintenance dose of 120 mg for a total of 10 times. The treatment period was 1 year, with a total of 19 sessions. Non‐response to BCG treatment was defined as follows: (1) disease recurrence within 6 months of non‐interrupted BCG treatment; (2) developing CIS within 1 year of BCG treatment.^13^ Disease recurrence was defined as tumor recurrence in the urinary tract in patients who underwent TURBT. Disease progression was defined as an increase in the TNM stage of the tumor; or an increase in the tumor grade.^14^ Recurrence‐free survival (RFS) was defined as the time from the initial TURBT to the date of disease recurrence or last follow‐up (December 2020). Progression‐free survival (PFS) was defined as the time from the initial TURBT to disease progression or last follow‐up (December 2020).

### 2.4 Statistical analysis

Statistical analyses were performed using the SPSS 26.0 statistical software. Non‐normal continuous variables were expressed as medians (interquartile range values), whereas categorical variables were expressed as frequencies (frequency). Nonparametric tests were used to analyze non‐normal continuous variables, while Pearson's chi‐square tests were used to analyze categorical variables. Univariable and multivariable logistic regression analyses were employed to determine predictors of BCG treatment response. The receiver operating characteristic (ROC) curve was used to determine the optimal cutoff values for the PLR, NLR, MLR, and SIRI in screening for BCG response in NMIBC patients based on the maximal Youden index. In addition, propensity score matching (PSM) was used to eliminate bias due to confounding factors between the low and high SIRI groups. The low and high SIRI groups were matched 1:1 using a match tolerance of 0.02 for the propensity score through caliper matching.^15^ The differences in RFS and PFS before and after PSM were compared between the two groups using the Kaplan–Meier method. The parameters included the mean survival time, median survival time, and 95% confidence interval (95% CI). A two‐tailed *p* value of <0.05 was considered statistically significant.

## 3 RESULTS

### 3.1 Clinicopathological features

A total of 540 NMIBC patients who underwent surgical treatment were included in this study, including 340 (63%) males and 200 (37%) females, with a median age of 63 (53–70) years. TNM staging identified 251 cases in the Ta stage and 289 in the T1 stage. 145 patients were identified as BCG non‐responders. The detailed clinical, pathological, and demographic characteristics are summarized in Table 1.

**TABLE 1:** Clinical characteristics of the patients according to the SIRI

Characteristics	Total (*n* = 540)	Low SIRI (*n* = 231)	High SIRI (*n* = 309)	*p* value
Age (years)	63 (53–70)	60 (52–69)	64 (55–71)	0.005
Sex (*n*, %)				
Male	340 (63)	139 (60.2)	201 (65)	0.246
Famale	200 (37)	92 (39.8)	108 (35)	
Smoking status (*n*, %)				0.002
Never	239 (44.3)	120 (51.9)	119 (38.5)	
Yes	301 (55.7)	111 (48.1)	190 (61.5)	
Diabetes (*n*, %)				0.132
No	478 (88.5)	210 (90.9)	268 (86.7)	
Yes	62 (11.5)	21 (9.1)	41 (13.3)	
Hypertension (*n*, %)				0.129
No	386 (71.5)	173 (74.9)	213 (68.9)	
Yes	154 (28.5)	58 (25.1)	96 (31.1)	
Tumor size (*n*, %)				0.003
<3 cm	295 (54.6)	143 (61.9)	152 (49.2)	
≥3 cm	245 (45.4)	88 (38.1)	157 (50.8)	
Tumor number (*n*, %)				0.002
Single	367 (68)	140 (60.6)	227 (73.5)	
Multiple	173 (32)	91 (39.4)	82 (26.5)	
Pathologic T stage (*n*, %)				0.326
pTa	251 (46.5)	113 (48.9)	138 (44.7)	
pT1	289 (53.5)	118 (51.1)	171 (55.3)	
Pathologic grade (*n*, %)				0.064
Low grade	279 (51.7)	130 (56.3)	149 (48.2)	
High grade	261 (48.3)	101 (43.7)	160 (51.8)	
BCG response (*n*, %)				<0.001
Non‐objective response	145 (26.9)	24 (10.4)	121 (39.2)	
Objective response	395 (73.1)	207 (89.6)	188 (60.8)	
Concomitant CIS (*n*, %)				0.003
No	504 (93.3)	224 (97)	280 (90.6)	
Yes	36 (6.7)	7 (3)	29 (9.4)	
LVI (*n*, %)				0.125
Absent	504 (93.3)	220 (95.2)	284 (91.9)	
Present	36 (6.7)	11 (4.8)	25 (8.1)	

Abbreviations: BCG, Bacillus Calmette‐Guerin; CIS, carcinoma in situ; LVI, lymphovascular infiltration; SIRI, systemic inflammation response index.

### 3.2 Potential predictive factors of BCG response

**FIGURE 2:**
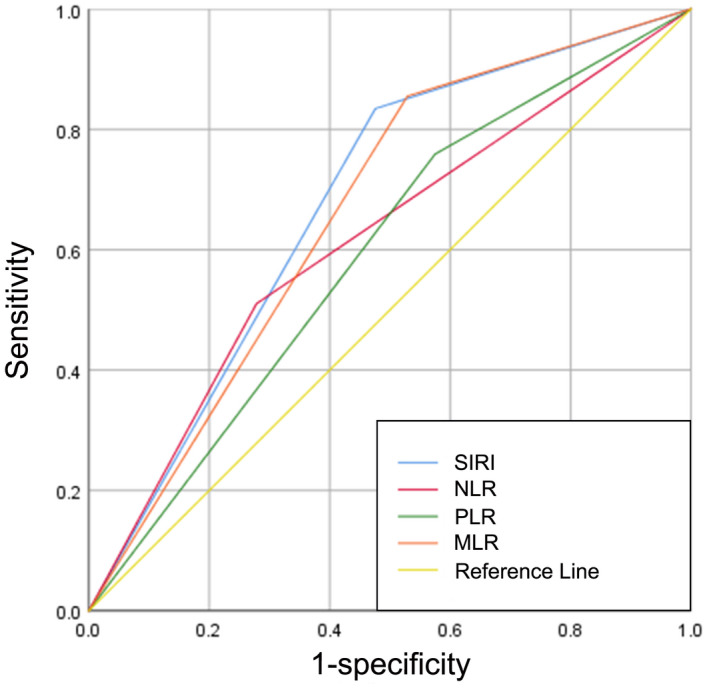
Comparison of the AUCs for PLR, NLR, MLR, and SIRI in predicting treatment response to intravesical BCG immunotherapy. AUCs, area under curves; BCG, Bacille Calmette‐Guérin; MLR, monocyte‐to‐lymphocyte; NLR, neutrophil‐to‐lymphocyte ratio; PLR, platelet‐to‐lymphocyte ratio; SIRI, systemic inflammation response index

145 (26.9%) patients were classified as BCG non‐responders, while 395 (73.1%) patients were identified as BCG responders during the follow‐up period. The optimal cutoff values for the PLR, NLR, MLR, and SIRI were 123.4398, 3.0435, 0.1995, and 0.785, respectively. Meanwhile, the area under the curves (AUCs) for PLR, NLR, MLR, and SIRI were 0.592 (sensitivity, Sen: 75.9%; specificity, Sep: 42.5%), 0.616 (sensitivity, Sen: 51%; specificity, Sep: 72.2%), 0.663 (sensitivity, Sen: 85.5%; specificity, Sep: 47.1%), and 0.679 (sensitivity, Sen: 83.4%; specificity, Sep: 52.4%), respectively (Figure 2). Notably, univariable analysis revealed that BCG non‐response was correlated with tumor size (*p* = 0.01), pathological T stage (*p* = 0.005), concomitant CIS (*p* = 0.006), PLR > 123.4398 (*p* < 0.001), NLR > 3.0435 (*p* < 0.001), MLR > 0.1995 (*p* < 0.001), and SIRI >0.785 (*p* < 0.001). Afterward, using the significant risk factors identified in the univariable analysis, a forward stepwise multivariable analysis identified independent predictors associated with BCG non‐response, including pathologic T stage (*p* = 0.015; OR = 1.776, 95% CI 1.164–2.711), MLR > 0.1995 (*p* = 0.015; OR = 2.229, 95% CI 1.172–4.238), and SIRI >0.785 (*p* = 0.001; OR = 3.139, 95% CI 1.701–5.792) (Table 2).

**TABLE 2:** Assessment of potential risk factors for BCG response

Characteristics	BCG responders (*n* = 395)	BCG non‐responders (*n* = 145)	Univariable			Multivariable		
			OR	95%CI	*p* value	OR	95%CI	*p* value
Age (years)	62 (53–70)	64 (55–71)	1.014	0.998–1.031	0.09			
Sex (*n*, %)			1.212	0.813–1.808	0.345			
Male	244 (61.8)	96 (66.2)						
Female	151 (38.2)	49 (33.8)						
Smoking status (*n*, %)			1.427	0.967–2.106	0.073			
Never	184 (46.6)	55 (37.9)						
Current or ex‐smoker	211 (53.4)	90 (62.1)						
Diabetes (*n*, %)			1.234	0.693–2.198	0.474			
No	352 (89.1)	126 (86.9)						
Yes	43 (10.9)	19 (13.1)						
Hypertension (*n*, %)			0.939	0.614–1.435	0.771			
No	281 (71.1)	105 (72.4)						
Yes	114 (28.9)	40 (27.6)						
Tumor size (*n*, %)			1.651	1.126–2.422	0.01	1.223	0.798–1.876	0.355
<3 cm	229 (58)	66 (45.5)						
≥3 cm	166 (42)	79 (54.5)						
Tumor number (*n*, %)			0.859	0.568–1.3	0.473			
Single	265 (67.1)	102 (70.3)						
Multiple	130 (32.9)	43 (29.7)						
Pathologic T stage (*n*, %)			1.745	1.18–2.58	0.005	1.776	1.164–2.711	0.015
pTa	198 (50.1)	53 (36.6)						
pT1	197 (49.9)	92 (63.4)						
Pathologic grade (*n*, %)			1.159	0.792–1.697	0.447			
Low grade	208 (52.7)	71 (49)						
High grade	187 (47.3)	74 (51)						
Concomitant CIS (*n*, %)			2.628	1.326–5.211	0.006	1.821	0.856–3.871	0.119
No	376 (95.2)	128 (88.3)						
Yes	19 (4.8)	17 (11.7)						
LVI (*n*, %)			1.215	0.582–2.537	0.604			
Absent	370 (93.7)	134 (92.4)						
Present	25 (6.3)	11 (7.6)						
PLR (*n*, %)			2.326	1.514–3.574	<0.001	1.436	0.872–2.364	0.155
≤123.4398	168 (42.5)	35 (24.1)						
>123.4398	227 (57.5)	110 (75.9)						
NLR (*n*, %)			2.7	1.823–4	<0.001	0.984	0.607–1.595	0.947
≤3.0435	285 (72.2)	71 (49)						
>3.0435	110 (27.8)	74 (51)						
MLR (*n*, %)			5.255	3.178–8.689	<0.001	2.229	1.172–4.238	0.015
≤0.1995	186 (47.1)	21 (14.5)						
>0.1995	209 (52.9)	124 (85.5)						
SIRI (*n*, %)			5.551	3.434–8.975	<0.001	3.139	1.701–5.792	0.001
≤0.785	207 (52.4)	24 (16.6)						
>0.785	188 (47.6)	121 (83.4)						

Abbreviations: BCG, Bacillus Calmette‐Guerin; CIS, carcinoma in situ; LVI, lymphovascular infiltration; MLR, monocyte‐to‐lymphocyte ratio; NLR, neutrophil to lymphocyte ratio; PLR, platelet to lymphocyte ratio; SIRI, systemic inflammation response index.

### 3.3 Impact of low and high SIRI on the RFS and PFS

**FIGURE 3:**
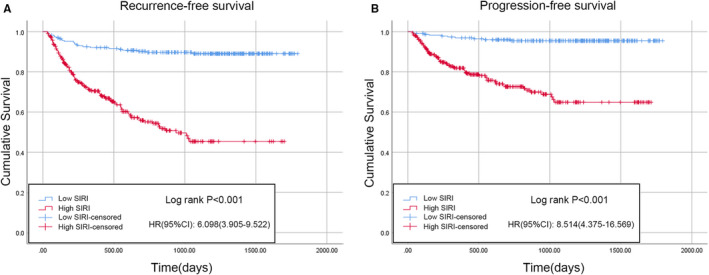
Kaplan–Meier curves and log‐rank tests were used to compare RFS (A) and PFS (B) between high SIRI and low SIRI groups before PSM. CI, confidence interval; HR, hazard ratio; PFS, progression‐free survival; PSM, propensity score matching; RFS, recurrence‐free survival; SIRI, systemic inflammation response index

**FIGURE 4:**
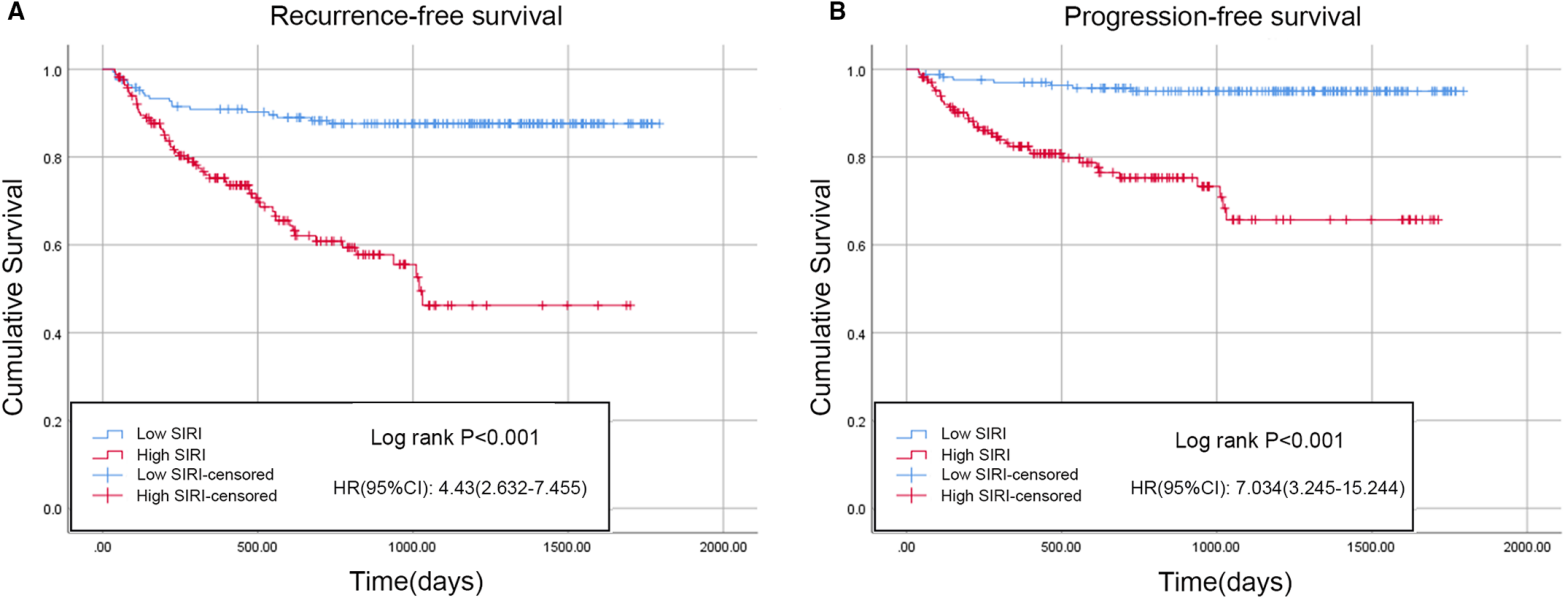
Kaplan–Meier curves and log‐rank tests were used to compare RFS (A) and PFS (B) between high SIRI and low SIRI groups after PSM. CI, confidence interval; HR, hazard ratio; PFS, progression‐free survival; PSM, propensity score matching; RFS, recurrence‐free survival; SIRI, systemic inflammation response index

According to the results of the logistic regression model, the PLR, NLR, MLR, and SIRI were associated with BCG treatment response in the univariable analysis, whereas only the SIRI and MLR were significantly correlated with BCG response in the univariable analysis; interestingly, the SIRI had a lower P value than the MLR. Therefore, Kaplan–Meier analysis was performed to analyze whether there were differences in RFS or PFS between patients with a low or high SIRI. There were 231 patients (42.8%) with a low SIRI and 309 patients (57.2%) with a high SIRI. Compared with low SIRI patients, those with a high SIRI were typically older, smoked more, had a higher tumor number, a larger tumor volume, and were more likely to develop concomitant CIS and BCG non‐response (Table 1). The Kaplan–Meier survival curves for the RFS and PFS of the two groups are depicted in Figure 3. Compared with the low SIRI group, the high SIRI group had significantly shorter median RFS (low SIRI, 1630.8 days [95% CI 1568.6–1693.1] vs. high SIRI, 990.5 days [95% CI 896.8–1084.2]; *p* < 0.001) (Figure 3A) and PFS times (low SIRI, 1726.8 days [95% CI 1685.8–1767.8] vs. high SIRI, 1261.6 days [95% CI 1174.4–1348.8]; *p* < 0.001) (Figure 3B). Furthermore, the results after PSM were as follows: A total of 334 patients (167 patients in each group) were included after PSM. The baseline characteristics of the two cohorts were comparable after PSM (Table 3). Consistent with the results of the Kaplan–Meier analysis, the high SIRI group had significantly shorter median RFS (low SIRI, 1602.5 days [95% CI 1523.3–1681.7] vs. high SIRI, 1046.4 days [95% CI 916.2–1176.6]; *p* < 0.001) (Figure 4A) and PFS times (low SIRI, 1719.2 days [95% CI 1668.4–1770.1] vs. high SIRI, 1290.0 days [95% CI 1173.1–1406.9]; *p* < 0.001) compared with the low SIRI group (Figure 4B).

**TABLE 3:** Clinical characteristics of the patients according to the SIRI after Propensity Score Matching

Characteristics	Total (*n* = 334)	Low SIRI (*n* = 167)	High SIRI (*n* = 167)	*p* value
Age (years)	61 (52.75–70)	61 (53–69)	62 (51–70)	1
Sex (*n*, %)				
Male	212 (63.5)	101 (60.5)	111 (66.5)	0.256
Female	122 (36.5)	66 (39.5)	56 (33.5)	
Smoking status (*n*, %)				0.912
Never	145 (43.4)	73 (43.7)	72 (43.1)	
Yes	189 (56.6)	94 (56.3)	95 (56.9)	
Diabetes (*n*, %)				0.5
No	294 (88)	149 (89.2)	145 (86.8)	
Yes	40 (12)	18 (10.8)	22 (13.2)	
Hypertension (*n*, %)				0.468
No	238 (71.3)	122 (73.1)	116 (69.5)	
Yes	96 (28.7)	45 (26.9)	51 (30.5)	
Tumor size (*n*, %)				0.511
<3 cm	178 (53.3)	86 (51.5)	92 (55.1)	
≥3 cm	156 (46.7)	81 (48.5)	75 (44.9)	
Tumor number (*n*, %)				0.633
Single	234 (70.1)	115 (68.9)	119 (71.3)	
Multiple	100 (29.9)	52 (31.1)	48 (28.7)	
Pathologic T stage (*n*, %)				0.444
pTa	163 (48.8)	85 (50.9)	78 (46.7)	
pT1	171 (51.2)	82 (49.1)	89 (53.3)	
Pathologic grade (*n*, %)				0.584
Low grade	175 (52.4)	90 (53.9)	85 (50.9)	
High grade	159 (47.6)	77 (46.1)	82 (49.1)	
BCG response (*n*, %)				<0.001
Non‐objective response	78 (23.4)	20 (12)	58 (34.7)	
Objective response	256 (76.6)	147 (88)	109 (65.3)	
Concomitant CIS (*n*, %)				0.078
No	312 (93.4)	160 (95.8)	152 (91)	
Yes	22 (6.6)	7 (4.2)	15 (9)	
LVI (*n*, %)				1
Absent	312 (93.4)	156 (93.4)	156 (93.4)	
Present	22 (6.6)	11 (6.6)	11 (6.6)	

Abbreviations: BCG, Bacillus Calmette‐Guerin; CIS, carcinoma in situ; LVI, lymphovascular infiltration; SIRI, systemic inflammation response index.

### 3.4 Combining SIRI and traditional clinical indicators to predict BCG response

**FIGURE 5:**
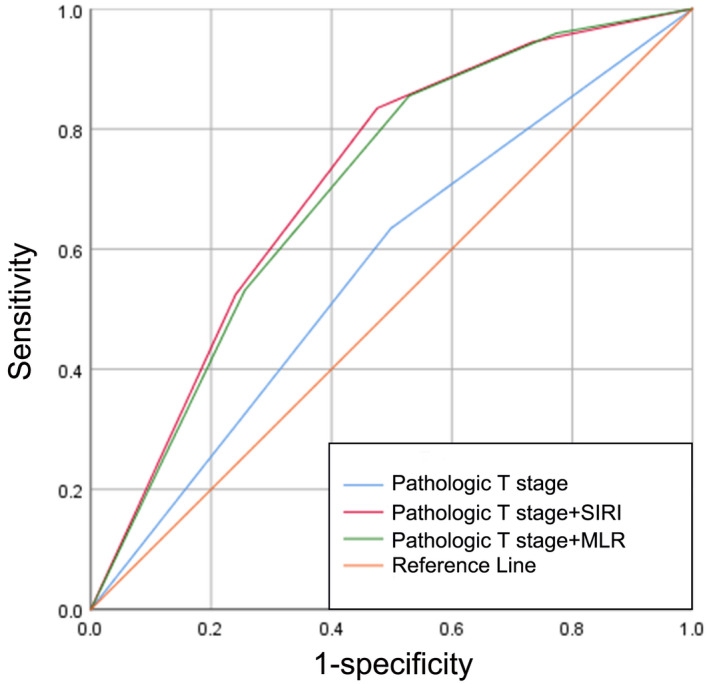
Comparison of the AUCs for pathological T stage, pathological T stage combined with the SIRI, and pathological T stage combined with the MLR in predicting BCG response in NMIBC patients. AUCs, area under curves; BCG, Bacille Calmette‐Guérin; MLR, monocyte‐to‐lymphocyte ratio; NMIBC, non‐muscle‐invasive bladder cancer; SIRI, systemic inflammation response index

The ROC curves of pathological T stage, pathological T stage combined with the SIRI, and pathological T stage combined with the MLR to predict BCG response are displayed in Figure 5, with AUCs of 0.568 (sensitivity, Sen: 63.4%; specificity, Sep: 50.1%), 0.711 (sensitivity, Sen: 83.4%; specificity, Sep: 52.4%), and 0.701 (sensitivity, Sen: 85.5%; specificity, Sep: 47.1%), respectively (Figure 5). As expected, the combination of pathological T stage and the SIRI demonstrated superior discriminative power in predicting BCG response compared with the other two groups.

## 4 DISCUSSION

In view of the extremely high recurrence rate of NMIBC, several guidelines recommend intravesical BCG therapy for high‐risk NMIBC patients after TURBT.^16^ Moreover, numerous prospective randomized trials have corroborated that BCG is effective in preventing tumor recurrence, tumor progression, and mortality.^17^, ^18^ Nevertheless, patients may still experience disease recurrence following BCG treatment. Therefore, it is crucial to discover effective predictors in clinical practice. In this study, the cutoff value of the SIRI was determined to be 0.785; the AUC for the SIRI was superior to that of other inflammatory markers (PLR, NLR, and MLR). In the univariable and multivariable analyses, a high SIRI value was positively associated with BCG non‐response, whilst survival analyses performed before and after PSM uncovered that a high SIRI may lead to poor RFS and PFS outcomes. Although the cutoff point for the SIRI in predicting the prognosis of different cancers varies, the results of our study are in line with earlier studies that reported that a high SIRI is an independent prognostic indicator for the recurrence and progression of malignant tumors.^12^, ^19^, ^20^

Current research established that inflammation is one of the hallmarks of tumor development and progression.^21^ In recent years, a huge body of evidence has shown that cancer growth, recurrence, and progression are influenced by systemic inflammation, thereby affecting patient survival.^22^ In some types of cancer, inflammatory conditions are present before the tissue becomes malignant, whereby the cell survival inflammatory environment may contribute to the development of cancer.^23^ Indeed, bladder cancer initiation and progression are associated with immune system interactions, and an inflammatory environment may promote tumorigenesis and progression.^24^ Some scholars have demonstrated that the changes in regulatory T cells and tumor‐associated macrophages in NMIBC patients were related to poor prognosis.^25^ Max Kates et al. found that intravesical BCG immunotherapy induced an increase in CD4+ T cell populations and was more effective than intravesical chemotherapy.^26^ Modulating cancer progression by governing the expression of certain inflammatory cytokines has been shown to be a potential therapeutic approach.^27^ Therefore, inflammation plays a crucial role in bladder tumor immunosuppression, proliferation, invasion, and metastasis.

The key role of neutrophils in the tumor immune microenvironment has lately become a research hotspot.^28^ Mutations in healthy cells are a prerequisite but not sufficient to cause tumorigenesis. However, inflammation plays an important role in initiating tumorigenesis by destroying healthy tissues, and neutrophils participate in this process.^29^ They are attracted to different organs via CXCR2 ligands, and their elastase‐derived and immunosuppressive abilities are both involved in tumorigenesis.^30^, ^31^, ^32^, ^33^, ^34^ Reactive oxygen species generated by neutrophils and angiogenic factors may also be implicated in tumorigenesis, progression, and metastasis.^35^, ^36^, ^37^ Monocytes are recruited throughout different stages of tumorigenesis and development.^38^, ^39^, ^40^ On the one hand, monocyte chemoattractant protein 1 (MCP‐1) exerts chemotactic activity on monocytes in various tumor models and is one of the key chemokines mediating monocyte/macrophage migration and infiltration.^38^, ^41^ On the other hand, lymphocytes play a crucial role in antitumor immunity and can induce tumor cell apoptosis through cytotoxicity.^42^ A decrease in lymphocytes can lead to immune disorders and impede the ability of the immune system to fight tumors. After activation of CD8^+^ T cells, effector CD8+ cytotoxic T lymphocytes (CTLs) infiltrate the core or invasive site of tumors and kill tumor cells.^43^

Mihai et al. described that peripheral blood NLR was associated with the risk of tumor invasion in patients receiving TURBT.^44^ In recent years, many scholars have reported that MLR is associated with the prognosis of different cancers.^45^, ^46^, ^47^ Ekaterina Laukhtina et al. found that a higher PLR may lead to lower rates of partial pathological and/or complete pathological response.^48^ Nonetheless, the above variables contain only two cell types, whereas the SIRI consists of three different cell types, which may result in superior predictions for bladder urothelial carcinoma patients. According to recent studies, the SIRI has been shown to have an outstanding predictive value in different cancers. Chao et al. found that a higher SIRI was associated with LVI, and further studies concluded that it was associated with survival time in cervical cancer patients.^49^ In another study conducted by Hua et al., SIRI was associated with progesterone receptor status through multivariable analysis, thereby affecting the occurrence of breast cancer.^50^ Qi et al. postulated that SIRI was associated with survival in pancreatic adenocarcinoma patients receiving chemotherapy.^51^ Pancreatic cancer patients with a higher SIRI may lead to poor prognosis.^51^ These findings are consistent with ours in that patients with SIRI >0.785 were more prone to be non‐responsive to BCG immunotherapy and had a worse prognosis.

Herein, multivariable analysis demonstrated that the pathological T stage, MLR, and SIRI were independent predictors for BCG non‐response. Another study found that a high MLR was associated with poor prognosis in NMIBC patients treated with BCG,^52^ which is consistent with our MLR results. However, we found that the combination of the SIRI and pathological T stage showed superior discriminative power in predicting BCG response than the MLR combined with the pathological T stage. The clinical significance of our study is the identification of a noninvasive, easily accessible, and reproducible prognostic biomarker for predicting BCG response, and the SIRI meets these parameters. For NMIBC patients treated with BCG immunotherapy with a high preoperative SIRI, more intensive follow‐up and monitoring are recommended. Furthermore, tumor T staging in conjunction with the SIRI can enhance prediction efficiency.

Although the results of this study are encouraging, the following limitations should also be taken into account. (1) This study was a single‐center retrospective study, and a large number of patients were excluded due to a lack of follow‐up data or other factors, making the study more susceptible to selection bias. (2) Only patients with urothelial carcinoma were included in this study; other pathological types of bladder cancer were excluded. (3) In this study, preoperative inflammatory indicators were selected. The dynamic fluctuations in the SIRI between time points before and after BCG treatment may provide further information on inflammation and immunity in NMIBC. Therefore, we aim to conduct follow‐up studies to validate our findings.^4^ In the future, further multicenter, large sample, prospective clinical trials are warranted to further corroborate our findings.

## 5 CONCLUSION

In short, the present study demonstrated that a high pretreatment SIRI is an independent risk factor for BCG non‐response and indicative of a poor prognosis in NMIBC patients and may serve as a potential marker for treatment response monitoring. Taken together, the combination of T stage and SIRI demonstrated robust performance in predicting the response to BCG immunotherapy in NMIBC patients.

## AUTHOR CONTRIBUTIONS

**Kun Ye:** Conceptualization (equal); data curation (lead); formal analysis (lead); investigation (lead); methodology (lead); software (lead); supervision (equal); validation (equal); writing – original draft (lead). **Ming Xiao:** Conceptualization (equal); data curation (equal); investigation (equal); methodology (supporting); supervision (equal); validation (equal); visualization (equal); writing – review and editing (equal). **Zitaiyu Li:** Data curation (supporting); investigation (supporting); methodology (supporting); software (supporting); supervision (supporting); validation (supporting). **Kancheng He:** Conceptualization (supporting); resources (supporting); software (supporting); supervision (supporting); visualization (supporting). **Jinhua Wang:** Conceptualization (supporting); formal analysis (supporting); validation (supporting); visualization (supporting). **Liang Zhu:** Conceptualization (supporting); data curation (supporting); formal analysis (supporting); software (supporting); supervision (supporting); visualization (supporting). **Wei Xiong:** Investigation (supporting); methodology (supporting); project administration (supporting); supervision (supporting); validation (supporting); visualization (supporting). **Zhaohui Zhong:** Conceptualization (equal); formal analysis (supporting); project administration (equal); resources (equal); supervision (equal); writing – review and editing (equal). **Yuxin Tang:** Conceptualization (equal); formal analysis (equal); investigation (equal); project administration (lead); resources (equal); supervision (lead); validation (lead); visualization (equal); writing – review and editing (lead).

## ETHICS STATEMENT

The study was approved by the Clinical Research Ethics Committee of The Second Xiangya Hospital of Central South University (XYEYY ‐2021 ‐K019) and adhered to the Declaration of Helsinki.

## Data Availability

The data that support the findings of the study are available from the correponding author upon reasonable request
